# Seeing shapes in seemingly random spatial patterns: Fractal analysis of Rorschach inkblots

**DOI:** 10.1371/journal.pone.0171289

**Published:** 2017-02-14

**Authors:** R. P. Taylor, T. P. Martin, R. D. Montgomery, J. H. Smith, A. P. Micolich, C. Boydston, B. C. Scannell, M. S. Fairbanks, B. Spehar

**Affiliations:** 1 Physics Department, University of Oregon, Eugene, OR, United States of America; 2 Code 7165, U.S. Naval Research Laboratory, Washington, DC, United States of America; 3 School of Physics, UNSW Australia, Sydney, Australia; 4 Department of Sciences and Mathematics, California State University Maritime Academy, Vallejo, CA, United States of America; 5 School of Psychology, UNSW Australia, Sydney, NSW, Australia; Ludwig-Maximilians-Universitat Munchen, GERMANY

## Abstract

Rorschach inkblots have had a striking impact on the worlds of art and science because of the remarkable variety of associations with recognizable and namable objects they induce. Originally adopted as a projective psychological tool to probe mental health, psychologists and artists have more recently interpreted the variety of induced images simply as a signature of the observers’ creativity. Here we analyze the relationship between the spatial scaling parameters of the inkblot patterns and the number of induced associations, and suggest that the perceived images are induced by the fractal characteristics of the blot edges. We discuss how this relationship explains the frequent observation of images in natural scenery.

## Introduction

In 1921, Herman Rorschach (1884–1922) published the images of ten inkblot patterns that were believed to unlock the hidden secrets of the human unconscious [1]. Promoted as a ‘psychological X-ray’, psychiatrists adopted the blots as a probe of mental health based on the phenomenon of pareidolia in which familiar patterns are perceived in stimuli when none actually exist. Rorschach patterns remain unrivaled in their application since quite possibly “no other psychological test has been administered to more millions of people throughout the world” [2]. Today, their role as a projective psychological tool has only historical value [3]. Instead, the percepts induced by the blots are interpreted as a signature of observers’ creativity [4]. In support of this view, artists ranging from the Surrealists to Andy Warhol have employed blot patterns as a vehicle to trigger their imaginations. The remarkable propensity of these simple shapes to induce such a rich variety of imagery, with up to 300 different percepts recorded for each blot [5], remains intriguing. If the pattern characteristics that stimulate this prolific visual activity can be identified and quantified, the resulting advances could potentially impact on diverse applications ranging from camouflage design to artificial vision.

A previous empirical investigation of pareidolia [6] was motivated by the observation that humans readily perceive and identify meaningful images in many naturally occurring but largely unstructured configurations ranging from clouds, rocks, and cracks in the ground to the surface of the Moon. All of these structures are fractal, featuring patterns that repeat at increasingly fine magnifications [7]. The study explored the relationship between the scaling characteristics of fractal patterns, as quantified by their fractal dimension *D* [7], and the patterns’ ability to evoke the perception of namable objects. Using computer-generated stimuli, fractals with low *D* values were found to elicit a higher ability to evoke the perception of namable objects. This was the same regardless of whether the fractal patterns contained only contours or filled-in regions. A more recent study showed that incorporating left-right symmetry (a central feature of inkblots) into computer-generated fractal stimuli further increased pareidolia [8].

When observers experience pareidolia, investigations of neural responses reveal that regions of the brain associated with object recognition are activated [8, 9]. For a diverse variety of patterns, ranging from fractals and overhead satellite imagery to magazine covers, ratings of ‘conspicuity’ (the ease of noticing images) elicited by these patterns were remarkably consistent within and between observers and remained so over a period of at least one year [10]. These results strongly suggest that perceived conspicuity is driven by pattern-inherent sensory factors common to all observers.

Consistent with this pattern-driven approach to perceived images, we present analysis showing that the number of induced namable images perceived in Rorschach patterns is related to fractal characteristics that occur at the edges of the blots. Our analysis, of course, does not address the idiosyncratic inter-individual variability in the type of images perceived in such patterns, but convincingly shows that the number of induced images can be related to the fractal scaling parameters. The results of our analysis also provide an explanation for the frequent appearance of recognizable imagery within natural objects such as clouds, rock faces and coastlines.

## Fractal analysis of the Rorschach inkblots

Historically, the technique used to generate the Rorschach inkblots has been shrouded in mystery. In particular, Rorschach’s artistic input has rarely been recognized [3]. After dripping black ink and water-color tints onto a sheet of white card, he then smeared the liquid using a pen before folding the card and pressing the two surfaces together [3]. Whereas the large-scale patterns of the resulting symmetric inkblot are therefore a consequence of the ‘painting’ style by which Rorschach consciously distributed the liquid across the sheet surface, the finer structure in the blot pattern emerged in the second stage of the process, as the liquid spread through the card fiber when held under pressure. Although governed essentially by the physical rules of fluid flow, Rorschach’s creativity in this second process materialized through his selection of the physical conditions dictating the flow. These conditions are regarded as crucial for shaping the intricate structure of the resulting blots. So crucial that the Rorschach Society only approved replica blots generated using Rorschach’s original tools and on days when the air humidity precisely matched the original conditions [2]. Today’s commercially-available Rorschach blots consist of scanned images of the original blots to avoid variations in blot appearance.

**Fig 1:**
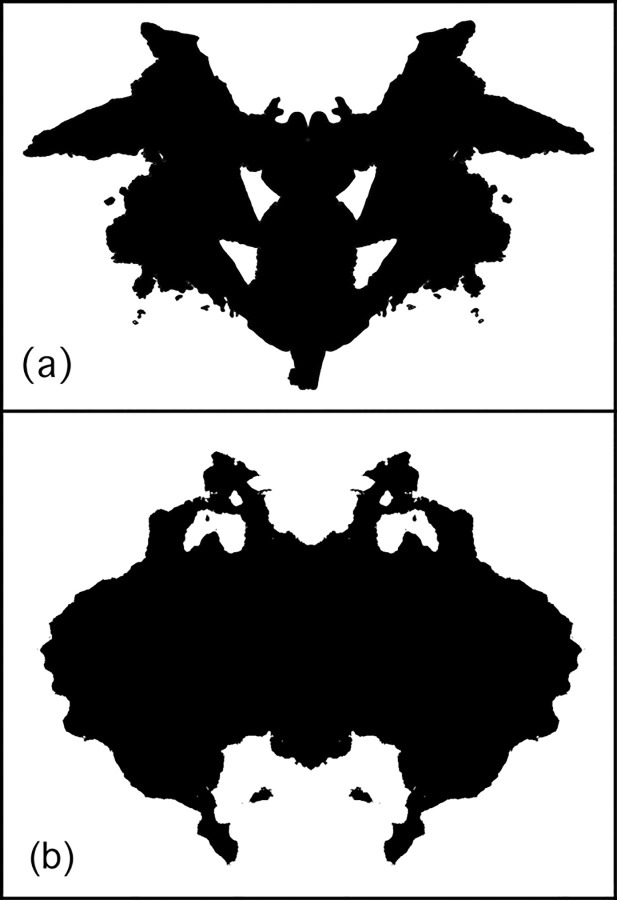
(A) Rorschach Blot One (with dimension *D* = 1.11), (B) a computer-generated fractal blot (*D* = 1.15).

Despite this apparently alchemic approach to the understanding of Rorschach’s blots, the procedure for generating the fine structure of the inkblots is analogous to that employed in many traditional studies of fluid morphology [11]. For example, in Hele-Shaw (HS) experiments, a fluid is injected under pressure though a porous medium and the pattern produced by the fluid boundary is known to be fractal, with roughness exhibited on many size scales [11]. This arises due to a competition between the driving fluid pressure and the local resistive forces in the porous medium. In the case of inkblots, the driving pressure gradually reduces as the blot spreads out, and the blot boundary stops spreading when the driving force becomes balanced by the local resistive pressure of the blot fibers. This resistive pressure varies locally, producing the spatial roughness of fractal patterns [11]. In Fig 1, we compare Rorschach Blot One with a computer-generated fractal blot (generated using a random midpoint displacement technique described elsewhere [12]) to demonstrate the visual similarity of their fractal features.

### Methods

**Fig 2:**
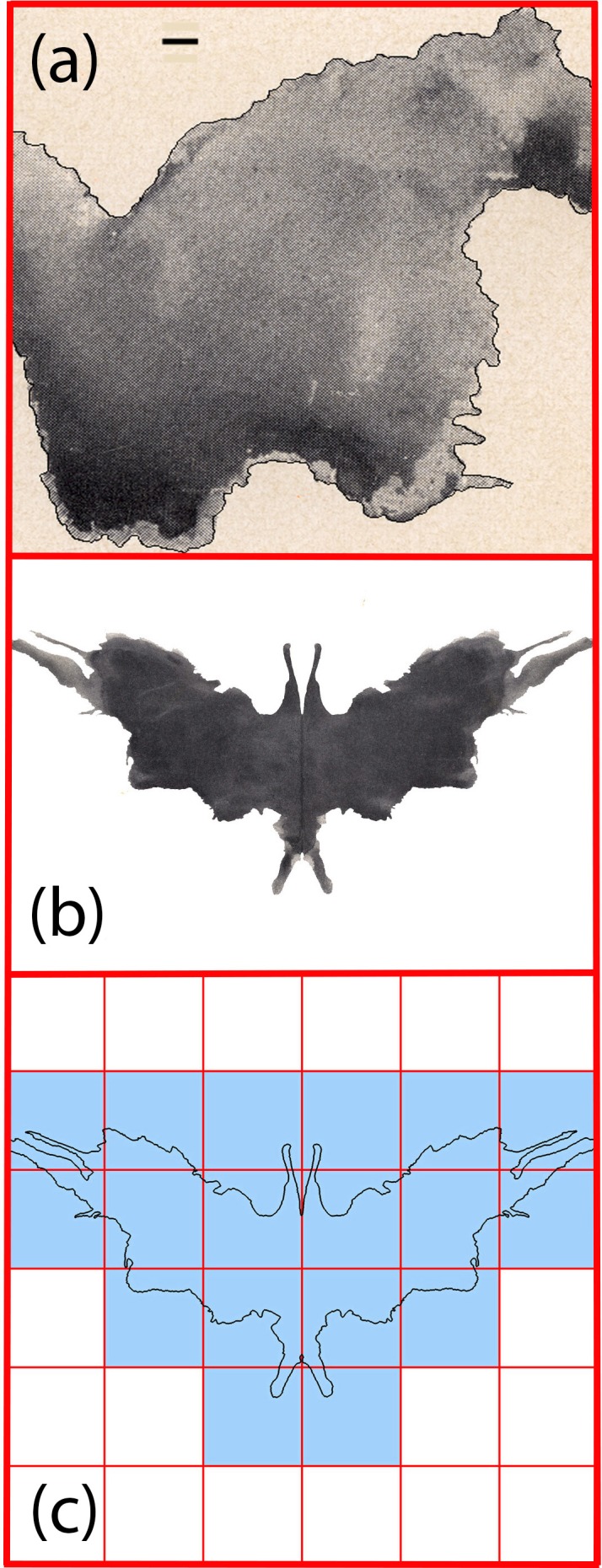
(A) A zoom-in on Blot Seven highlighting the extracted boundary with a black line. The scale bar corresponds to 1.6mm (19 pixels in the scanned image). (B) Rorschach Blot Five of width 17.5cm. (C) A schematic representation of the box-counting technique applied to Blot Five’s boundary. The box size shown represents the largest box size analyzed.

To quantify the fractal scaling properties of Rorschach’s blots, we performed an analysis on the boundaries of the ten blots. The procedure is demonstrated in Fig 2. First, the blot was scanned at 300dpi and the boundary between the regions of ink absorption and the unstained card was extracted using an edge detection computer analysis [13] with an accuracy of 0.5mm (corresponding to 6 pixels in the scanned image).

**Fig 3:**
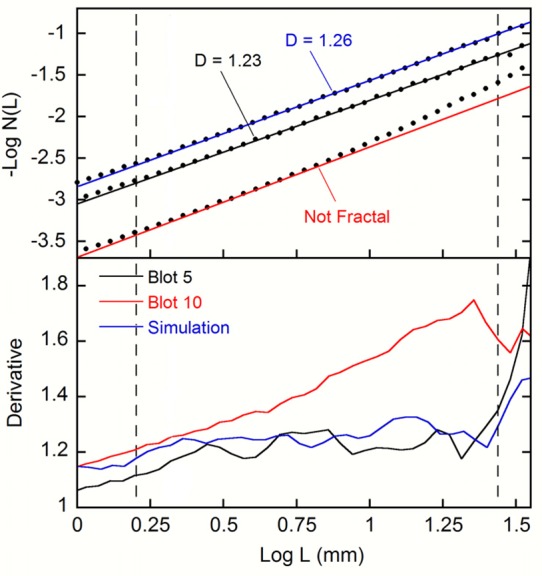
Top three traces: Box-counting analyses applied to Rorschach Blot Five (black), Rorschach Blot Ten (red), and a computer-generated fractal boundary (blue). Bottom three traces: the derivatives of the three scaling plots (see text for details).

A traditional technique for measuring a boundary’s dimension fractal *D*, referred to as the box-counting method, is shown in Figs 2 and 3. The boundary’s *D* value describes how the patterns occurring at different magnifications combine to build the resulting fractal shape [14]. For Euclidean shapes, dimension is described by the familiar integer values − for a smooth line (containing no fractal structure) *D* has a value of 1, while for a completely filled area (again containing no fractal structure) its value is 2. For the repeating patterns of a fractal line, *D* lies between 1 and 2, and, as the repeating structure covers more space, its value moves closer to 2. To extract *D*, the boundary pattern of the blot was covered with a computer-generated mesh of identical squares (or ‘boxes’). The number of squares, *N*(*L*), that contain part of the pattern was then counted (i.e. the shaded boxes in Fig 3(C)), and this count was repeated as the size, *L*, of the squares in the mesh was reduced. *N*(*L*) gives a measure of the space coverage of the pattern, and reducing the square size is equivalent to looking at this coverage at finer magnifications. For fractal behavior, *N*(*L*) scales according to the power law relationship *N*(*L*) *~ L*^*-D*^, where 1 < *D* < 2 [14].

### Results

The above power law generates the scale-invariant properties that are central to fractal geometry and manifests itself as a straight line in the scaling plot of log *N*(*L*) versus log *L*, as shown in Fig 3 for Rorschach Blot Five. An automated procedure was used to determine the scaling range that generates the best linear fit to the data [15]. This was achieved by varying the range of data points included in the fit in order to minimize the variance of the data from the fit line. The quality of the fit was quantified by the coefficient of determination *R*^*2*^, which increases with the quality of the fit and lies in the range 0 to 1 [15]. The black fit line for Blot Five is described by *D* = 1.23 and *R*^*2*^ = 0.9995.

The vertical dashed lines represent the coarse and fine cut-offs determined by the fitting procedure. The coarse scale cut-off corresponds to the measurement limit originating from the reduced counting statistics that occur when the number of boxes in the grid becomes too few [16]. For box sizes larger than 2.7cm, there are insufficient boxes to distinguish the fractal boundary from that of a filled space: the gradient then increases toward *D* = 2 because all the boxes in the mesh become occupied by the boundary pattern. This coarse scale measurement cut-off cannot be improved upon because it is linked to the blot size. However, this does not limit our study because patterns larger than this scale are expected to be dominated by Rorschach’s ‘painting’ process rather than the fluid-induced fractals.

The observed fine-scale cut-off occurs at a much larger scale (19 pixels) than the fine scale measurement cut-off (6 pixels, set by the accuracy of the edge extraction process). To emphasize this difference, the scale bar included in Fig 2(A) represents the box size at the observed cut-off (1.6mm). The observed fine scale cut-off therefore represents a physical limit of the fractal generation process and is a characteristic of HS-type experiments [11]. Although boundary structure exists below this scale, it is no longer described by a fractal power-law behavior.

**Fig 4:**
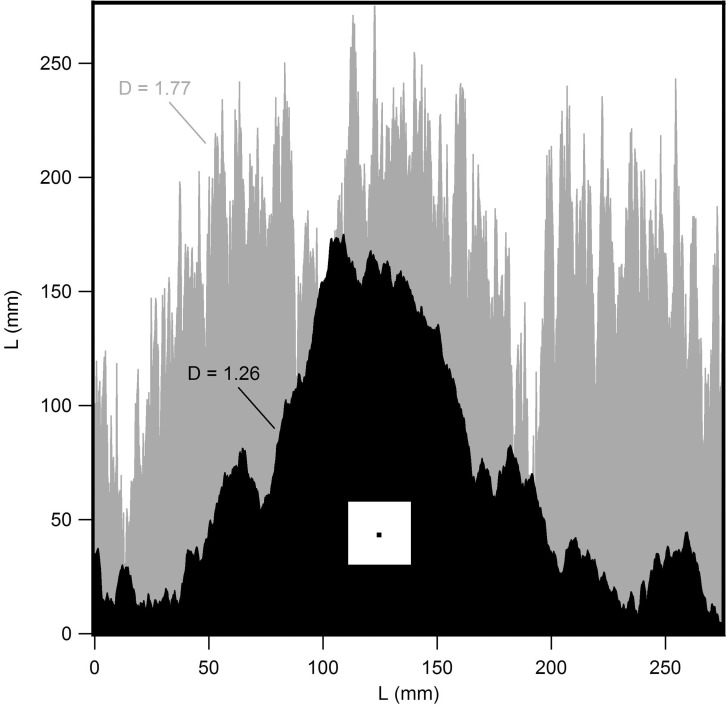
Computer-generated fractal boundaries. The black pattern has a *D* value of 1.26 that closely matches that of Rorschach Blot Five, while the grey pattern has a much higher *D* value of 1.77. The black and white squares indicate the smallest and largest box sizes used in the fractal analysis of these boundaries.

Fig 3 also includes the scaling plot achieved when the box-counting method was applied to the computer-generated fractal boundary shown in Fig 4. This boundary was generated using a Fourier transform technique [17] and has a *D* value (1.26) similar to that of Blot Five. For comparison, the blue fit line was generated over the same scaling range as Blot Five and is quantified by *R*^*2*^ = 0.9997. Whereas the *R*^*2*^ value of the Blot Five fit matches this down to three decimal places, the fit line for Blot Ten over the same scaling range is quantified by a *R*^2^ value which matches it to only two (0.9976), indicating that Blot Ten does not follow the fractal power law behavior as closely. When the fitting procedure for Blot Ten was allowed to select the data points which minimize *R*^*2*^ (resulting in the red line quantified by *R*^*2*^ = 0.9992), the scaling range of *L* is significantly less than a factor of ten. Although the definition of fractals does not feature a minimum size range for scaling [7], power laws typically require a minimum of one order of magnitude to be detected with confidence.

To emphasize Blot Ten’s deviation from fractal behavior, the derivatives of the three scaling plots are plotted below the data lines in Fig 3. In each case, the derivative was calculated using a linear fit to the four nearest neighbors to either side of each data point (i.e. nine points were used to determine each of the derivative values). The derivatives for Blot Five (black trace) and the computer-generated fractal (blue trace) are horizontal within the region between the vertical dashed lines (the *D* values of which are plotted on the left-hand axis), while the derivative for Blot Ten (red trace) increases gradually. These behaviors don’t extend right up to the cut-off lines because of the 9 point calculation (for example, a derivative value plotted on the cut-off line will be influenced by 4 points within the fractal region and 4 points outside). Note also that the ‘bumps’ in the derivative plots are an intrinsic feature of fractals (as evidenced by their appearance in the data of the computer-generated fractal) and result from statistical variations.

Blot Five is one of five Rorschach blots formed by using only black ink. The fractal scaling properties of all five of these black blots are confirmed by the box-counting analysis and also by an independent technique known as the ‘coastline method’ [14]. The *D* values of the five blots are: Blot One (*D* = 1.11), Four (1.22), Five (1.23), Six (1.17) and Seven (1.13). This *D* range of 1.1 to 1.3 matches the values measured in the HS experiments [11]. In the HS experiments, differences in the measured *D* values result from variations in applied pressure and fluid viscosity, and we interpret the different *D* values of the five blots in terms of similar variations in Rorschach’s blot generation process. Within this picture, it is interesting to note that air humidity influences the fluid evaporation rate, leading to variations in the driving pressure of the spreading ink. This is consistent with the traditional belief that weather conditions influenced the formation of the fine-structure patterns in the blots [2].

**Fig 5:**
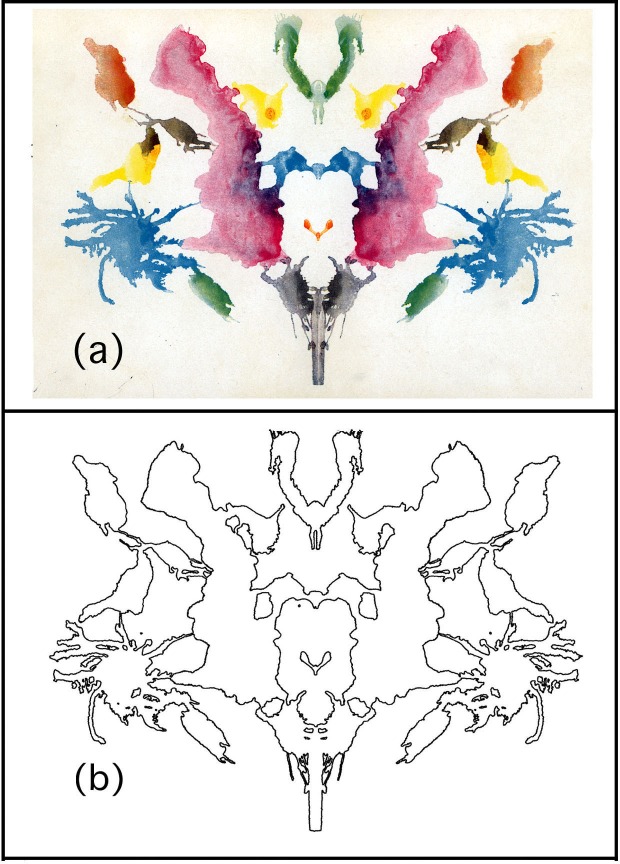
(A) Rorschach Blot Ten, (B) the boundaries of the blot edges.

The remaining five Rorschach blots are composed of several regions of different colors formed from black ink and water-color tints. The scaling plots of these colored blots are similar to the Blot Ten data shown in Fig 3. Note that, as with the black blots, the boundary of the multi-colored blots corresponds to the edge between stained and unstained blotting paper (i.e. color is ignored). The physical origin for the lack of fractality of the colored blots lies in the fact that the boundaries of the colored sub-regions are fractal but with different *D* values to each other (presumably due to the variations in ink fluidity and applied pressure). For example, the *D* values of the orange and blue regions of Rorschach Blot Ten are 1.1 and 1.41 respectively (see Fig 5). The scaling properties of a pattern composed from two individual fractals depends on factors such as the relative densities of the two patterns, the scaling behavior of boxes containing both patterns, and the difference in their *D* values [18]. In particular, for patterns such as the Rorschach blots, the large difference in *D* values for the sub-regions is expected to prevent fractal scale invariance of the combined pattern, as observed in the scaling plot shown in Fig 3 for Rorschach Blot Ten. Although the scaling plots of the colored blots show that their boundaries can’t be quantified by a simple box-counting dimension, we note that a multi-fractal analysis [19] might reveal additional information regarding their scaling characteristics.

## Images perceived in Rorschach inkblots

**Fig 6:**
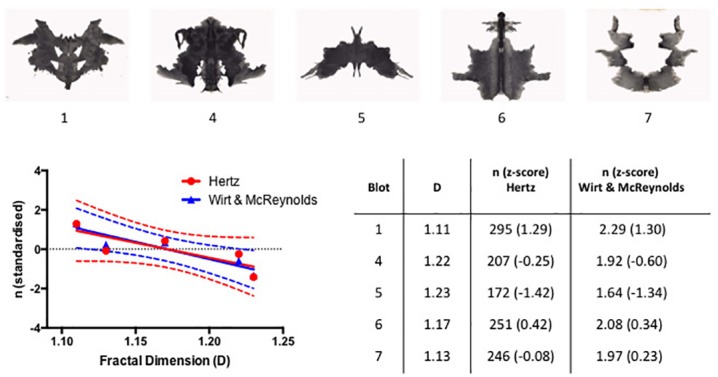
Number of percepts, *n*, induced by the five black blots plotted as a function of the *D* value of their boundaries. The inset tabulates the *D* values of the five blots along with the corresponding data from Hertz [5] and Wirt and McReynolds [21] respectively. For the purpose of direct comparison, the raw *n* scores have been standardized as z-scores and are shown in parentheses. The horizontal dotted line represents the mean score for each of the two sets of data. The red and blue lines are linear fits to the respective data and the dashed lines represent the boundaries of 95% confidence intervals. Note that three of the blue data points (for Blots 1, 5 and 6) are obscured by the equivalent red points.

A key question in our analysis concerns the relationship between the fractal properties of the blot boundaries and the number of images perceived when observing the blots. To address this, we re-visited two original empirical investigations concerning the number of responses to individual Rorschach blots. The first was the compilation of responses for each of the Rorschach blots conducted by psychologist Marguerite Hertz [20]. As the founding member of the Rorschach Institute, Hertz pioneered standardized scoring of the inkblot tests in the 1930s and produced the Frequency Tables for Scoring Rorschach Responses. These tables were created by meticulous cataloguing of different percept types (e.g. an image of a bat, a person, etc.) for each of the ten Rorschach blots generated by a cumulative sample of *N* = 1050 subjects aged 11–19 [5]. For the purpose of our analysis, we simply counted the number of different percept types reported for the five black blots and plotted these empirically determined values *n* against their respective *D* values obtained from our analysis (Fig 6, red symbols). Note that, just as *D* quantifies the boundary of the whole blot, so too *n* quantifies the number of percepts types induced by the whole boundary, and does not include percepts induced by component regions of the blots. The data reveal a decreasing trend indicating the importance of *D* in observing the number of induced percepts.

A strikingly similar trend can also be observed with the second independent empirical sample of percepts for each Rorschach blot, reported by Wirt and McReynolds in 1953 [21]. The total number of responses is one of the standard variables in an administration of the Rorschach test (the so called Rorschach variable “*R”*) and Wirt and McReynolds were concerned with the extent to which this measure is reliable, reproducible and consistent from one testing occasion to another. For that purpose, they determined the mean and standard deviation of the total number of responses for each blot in the samples of normal (*N* = 76), neurotic (*N* = 32) and schizophrenic (*N* = 50) subjects. The blots were then divided into two groups of five blots to determine whether the responses to the two groups were similar. Correlations between the scores for the two groups of blots equaled 0.88, 0.91 and 0.77 for the normal, neurotic and schizophrenic sample respectively, indicating high reliability (a score of 1 would represent a perfect correlation). While the relatively high reliability of the total number of responses is encouraging in itself, we took the opportunity to compare the reported number of total responses for each of the five black blots to those estimated based on the responses compiled by Hertz [5].

We note that the values recorded by Wirt and McReynolds represent the average number of percepts one person would report per blot in one administration of the test. More precisely, each value refers to the average number of responses given to the question “What might this be?” for each individual blot. On average, one person would see from one to three shapes in each blot, totaling 20 to 30 for the entire test. Thus, the *n* values for the Wirt and McReynolds test are much smaller than those from Hertz data, which are based on the cumulative number of percept types reported by more than 1000 participants. The Hertz numbers indicate the variability of responses on a group level while the Wirt and McReynolds numbers are to some extent an indicator of variability on the level of one individual. Despite these differences in recording *n*, the standardized z-scores for the average number of total responses reported by Wirt and McReynolds, plotted as blue symbols in Fig 6, closely follow the same inverse relationship between the *n* and *D* values found in the Hertz data.

## Images perceived in computer generated fractals

Naturally, the boundary’s fractal structure is not the only cause of the large number of percept induced by the black blots–other likely factors include the blot’s (left-right) symmetry [8] and also shading caused by variations in the ink’s opacity in the regions enclosed by the boundaries. One way to isolate the contribution of variations in fractal dimension is to investigate the perceived images in computer generated fractal patterns varying solely in *D*. A second motivation for using computer generated stimuli is that the blots study was inevitably limited to just ten stimuli. By extending the studies to computer stimuli, we investigated responses to 24 additional images.

### Participants

For this study, 23 first year Psychology students from the University of New South Wales were used (*N*_*male*_ = 10, *N*_*female*_ = 13) with an average age of 18.9 years. The students received extra course credit in exchange for their participation and had normal or corrected to normal vision. Prior to the start of the experiment, all participants signed an informed consent form. All procedures and protocols were approved by the University of New South Wales Human Research Ethics Approval Panel (HREAP C).

### Materials

**Fig 7:**
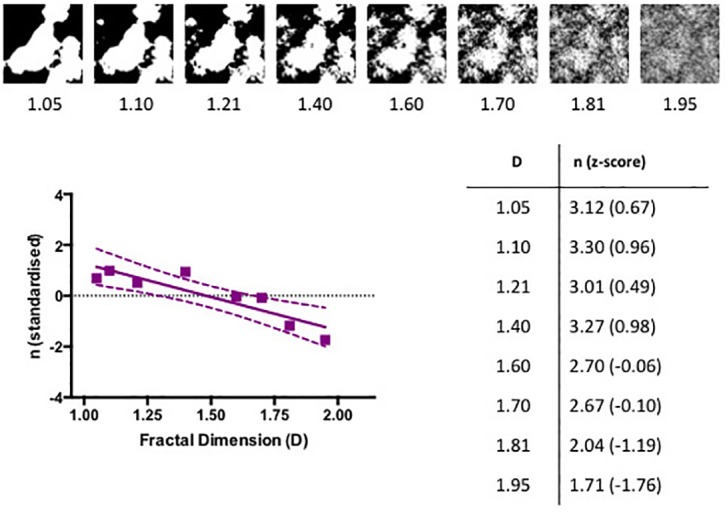
The standardized number of reported percepts, *n*, induced by the computer-generated fractal patterns plotted as a function of their *D* value. One set of computer-generated images is illustrated above the graph. The dotted lines represent the boundaries of 95% confidence intervals. The inset tabulates the *D* values of the images along with the corresponding average number of reported perceived shapes.

Three different ‘seed’ patterns were used to generate fractal images using a Fourier transform technique reported elsewhere [22]. Each seed set featured 8 images varying in *D* from 1.05 to 1.95. All images were 512 by 512 pixels wide and had the same mean luminance and contrast. One set of images is illustrated in the top section of Fig 7.

### Procedure

Prior to data collection, participants received printed versions of images showing the variations in *D* and performed 4 practice trials. The study was conducted in a room with controlled lighting and participants were placed 60cm away from the monitor. All stimuli subtended a visual angle of 12 by 12 degrees and were centered against a uniform grey background.

The presentation of each of the images was randomized and each image was presented once and for a duration of 10 seconds. Before each trial, a fixation cross appeared for 500 milliseconds. After each image was shown, participants were asked how many shapes they could discern in the image. The response options included “none”, “1 or 2 shapes”, “3 or 4 shapes”, “5 or 6 shapes” or “7 or more.”

### Results

Before analysis, the answers were converted to numerical values by taking the average of the numbers in each choice except for “none” which was given “0” and “7 or more” which was given “7.5”. The reported number of perceived shapes, averaged across the three different sets of images, is shown in Fig 7. We then applied the standard statistical procedure, Analysis of Variance (ANOVA), to the data. One-way repeated measures ANOVA revealed the significant effect of *D* on the number of reported shapes (*F*_*2*.*925*,*64*.*34*_ *=* 3.17, *p* = 0.03).

The relationship between *D* and *n* observed for the Rorschach blots and our computer-generated fractal patterns is also consistent with a previous study of computer-generated fractal patterns [6] and confirms that low *D* fractal boundaries provide the best stimulus for induced associations with namable objects. Our study confirms the effect for an extended range of *D* and for much finer interval steps between different *D* values. More crucially, the previous study simply displayed groups of four images with different *D* values and recorded the percentage of times each image was chosen over the others based on which image induced more precepts. In contrast, our experiment recorded the number of percepts induced by each image, allowing a direct comparison with the original blot tests.

## Discussion

Fractal dimension plays a crucial role in determining a pattern’s visual appearance. Returning to the scaling behavior of Fig 3, *D* corresponds to the gradient of the scaling plot. A high *D* value is therefore a signature of a large *N*(*L*) value at small *L* and reflects the fact that many small boxes are being filled by fine structure. This can be seen, for example, for the two computer-generated fractal boundaries shown in Fig 4. The fine features play a more dominant role for the *D* = 1.77 pattern than for the *D* = 1.26 pattern. The fine feature content for the *D* = 1.26 pattern is much closer to that of Blot Five (*D* = 1.23).

Because of this relationship between *D* and fine structure content, *D* is a well-established tool for quantifying fractal complexity [7, 14]. Traditional measures of visual patterns quantify complexity in terms of the ratio of fine structure to coarse structure. *D* goes further by quantifying the relative contributions of the fractal structure at all the intermediate magnifications between the coarse and fine scales. Previous psychophysical experiments performed on fractal patterns confirm that raising the *D* value increases its perceived complexity [23–25]. Thus, the increase in number of induced percepts between the different blots might be linked to a reduction in their fractal complexity.

In addition to the dependence of *n* on *D*, the importance of the fractal boundary for inducing percepts is further highlighted by the fact that the non-fractal, multi-colored blots induce fewer percepts (*n* = 140 to 170) than their fractal, black counterparts (*n* = 170 to 300) [5]. We note, however, that there may be additional causes for this drop in *n*. In particular, for the multi-colored blots, the component regions (each with a distinct color) might visually dominate over the whole blot. The boundary shapes of these component regions would then predominantly determine the percepts rather than the whole blot’s boundary. This would result in a lower *n* because *n* quantifies only percepts induced by the whole blot. This effect is consistent with findings suggesting the powerful role of color on image segmentation [26].

Although the *n* values are smaller for the colored blots than their black counterparts, they nevertheless still have significant magnitudes. In our current study, we focused on the box-counting dimension *D* because of the range of previous psychophysical experiments that linked the visual properties of fractals to *D* [6, 17, 22–25, 27–37]. However, as noted earlier, future studies should explore if other scaling parameters revealed by a multi-fractal analysis can explain the percepts induced by the colored blots.

**Fig 8:**
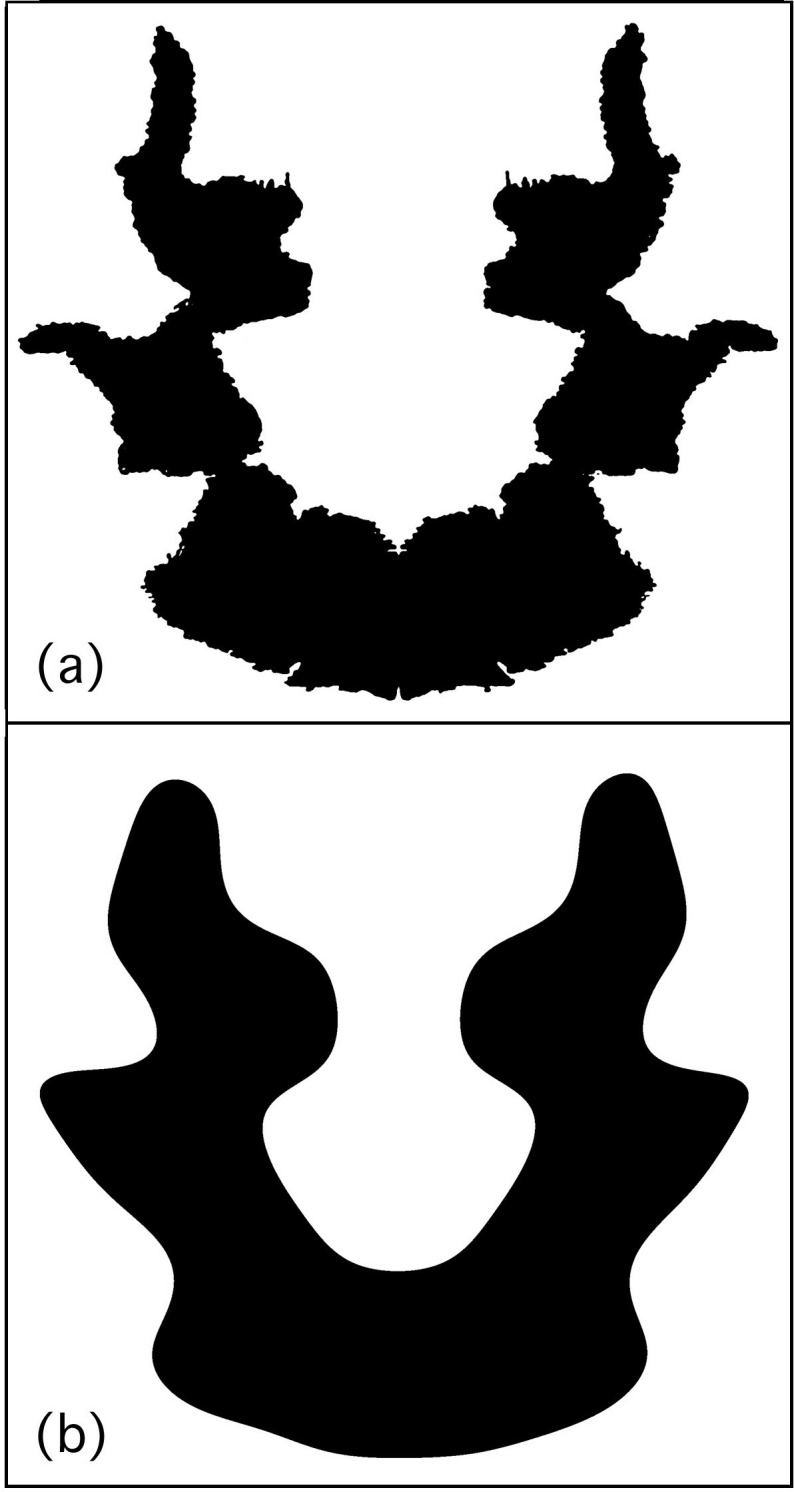
(A) Rorschach Blot Seven, (B) Rorschach Blot Seven with the fractal features removed.

It is interesting that the fractals play such an important role given that the structure is limited to size scales smaller than approximately 2.5cm (patterns larger than 2.5cm weren’t generated by the fractal ink diffusion process but by Rorschach’s ink smearing actions prior to pressing). In Fig 8, we therefore demonstrate the visual importance of the fractal structure by eliminating this structure from the boundary of Rorschach Blot Seven by Fourier transforming the blot image and removing spatial frequencies corresponding to the fractal scaling regime (2-25mm). This process clearly impacts on the visual perception of the blot.

**Fig 9:**
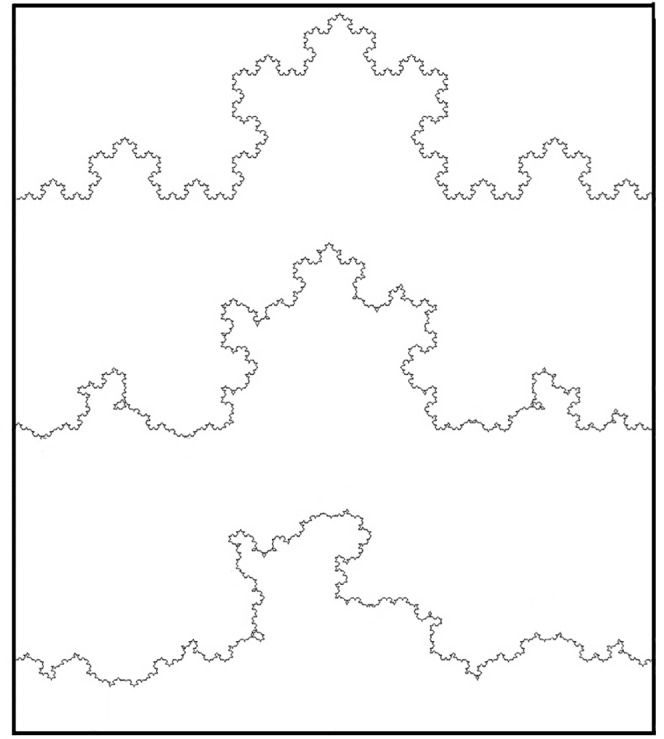
Koch curves quantified by *D* = 1.26. The exact fractal (top trace) is morphed into the statistical fractal (bottom trace). See the text for details.

The human visual system’s ability to detect fractal characteristics based on only a limited magnification range has been highlighted in the previous psychophysical experiments [6, 17, 22–25, 27–37]. This remarkable sensitivity to fractals is perhaps not surprising given that many of the physical fractals that define our daily visual environment exhibit fractal characteristics over limited magnification ranges [38]. This relationship with nature highlights another crucial factor for inducing perceived images—the statistical quality of the fractal pattern. Fig 9 shows a fractal pattern based on the Koch curve, which has a similar *D* value (1.26) to the Rorschach Blot Five of Fig 2 and the low *D* computer-generated fractal of Fig 4. In this figure, we morph the ‘exact’ fractal (where the patterns repeat exactly at different magnifications) into the more natural-looking ‘statistical’ fractal (in which the statistical qualities of the fractal repeat at different magnifications) by introducing random variations into the fractal pattern as follows. The probability *p* for a ‘spike’ on the curve to be pointing up or down changes for the three curves in Fig 9. For the traditional Koch curve *p* = 0, corresponding to zero probability of having a spike pointing down. For the second image *p* = 0.25, so most of the spikes are pointing up. For the third curve, *p* = 0.5, corresponding to a 50% chance of pointing up or down. In each case, the spatial distribution of the up and down spikes is random.

These random variations preserve the fractal scaling properties such as *D* while removing the artificial appearance of the exact Koch pattern, allowing the emergence of a more ‘organic’ visual character that we hypothesize enhances the pattern’s ability to induce associations with namable objects and/or recognizable imagery. Integration of random variations with fractal scaling of *D* = 1.1 appears to be optimal. We note that purely random white noise also has the capacity to induce percepts [8]. However, because white noise is characterized by *D* = 2 [39], the data of Figs 6 and 7 suggest that the lower *D* values (corresponding to pink noise [39]) will induce more percepts than purely random patterns.

Natural objects whose fractal characteristics might be expected to induce recognizable images include rock faces, coastline patterns, clouds and craters on the Moon. In particular, many clouds have fractal boundaries quantified by *D* = 1.3 (similar to the Rorschach blots) and are well-known for inducing percepts in our daily lives. Biological examples of fractals that induce associations with namable objects include animal markings [40], a phenomenon that inspired research of fractal camouflage [41]. Fractal-induced imagery is also evident in artworks. In particular, the Surrealists developed several techniques to produce patterns that induce imagery. A prime example is Oscar Dominguez’s technique, decalcomania [42], for which he painted a surface, pressed a sheet of paper down on that surface and then pulled it off. The infusion of air into the paint as the two layers were separated generated a fractal process called viscous fingering [43]. Dominguez described his resulting patterns as “unequalled in [their] power of suggestion”, emphasizing the propensity of these simple fractal patterns for triggering a striking variety of imagery. The Abstract Expressionist Jackson Pollock’s poured paintings are composed of fractals with *D* values that increased from 1.1 to 1.7 over the decade 1943–1952 [32, 44]. Intriguingly, Pollock seems to have been aware that his drive towards higher complexity paintings would reduce the number of induced precepts: “I try to stay away from any recognizable image; if it creeps in I try to do away with it… I don’t let the image carry the painting… It’s extra cargo—and unnecessary” [45].

## Conclusions

Analysis of Rorschach inkblots provides an appealing framework for understanding the rich variety of visual associations induced by fractal patterns spanning psychology, art and nature. In each case, the repetition of structure at increasingly fine magnifications generates the visual complexity necessary to induce the perceived images. Within this fractal model, low *D* fractals provide the optimal distribution of spatial frequencies to induce the images. In contrast, the dominance of fine structure in high *D* fractals appears to reduce the perception of recognizable images. The ability to tune the number of percepts by adjusting the *D* value of computer-generated fractals has huge potential for both the visual arts and visual sciences. Given the recent emphasis on the connection between the images induced by the blots and the observer’s creativity [4], it is intriguing to note that self-reported creative people prefer to look at fractals with higher *D* values [46]. It would therefore be interesting to investigate how the *n* vs *D* dependence identified in Figs 6 and 7 is affected by observers’ creativity. Another potential focus for future investigations concerns the relationship between percepts and aesthetics. We note that a previous study of the fractal boundaries of blots focused on their aesthetics [47]. However, the blots were analyzed after undergoing filtering for red, blue and green colors. This difference between their images and our unfiltered ones prevents a comparison between the two studies.
